# 3D virtual pre-operative planning may reduce the incidence of dorsal screw penetration in volar plating of intra-articular distal radius fractures

**DOI:** 10.1007/s00068-021-01800-2

**Published:** 2021-10-08

**Authors:** Jasper Prijs, Bram Schoolmeesters, Denise Eygendaal, Jean-Paul P. M. de Vries, Paul C. Jutte, Job N. Doornberg, Ruurd L. Jaarsma, Frank F. A. IJpma, on behalf of the Traumaplatform 3D Consortium

**Affiliations:** 1 Department of Orthopaedic and Trauma Surgery, Flinders University and Flinders Medical Centre, Adelaide, Australia; 2 Department of Orthopaedic Surgery, University of Amsterdam, Amsterdam University Medical Centres, Amsterdam, The Netherlands; 3 Department of Surgery, University of Groningen, University Medical Center Groningen, BA13, Hanzeplein 1, 9713 GZ Groningen, The Netherlands; 4 Department of Orthopaedic Surgery, University Medical Center Groningen, Groningen, The Netherlands; 5 Department of Orthopaedics and Sports Medicine, Erasmus University Medical Center, Rotterdam, The Netherlands

**Keywords:** 3D, Virtual planning, Virtual surgical planning, Dorsal screw penetration, Distal radius fracture

## Abstract

**Purpose:**

To evaluate the effect of three-dimensional virtual pre-operative planning (3DVP) on the incidence of dorsal screw penetration after volar plating of distal radius fractures.

**Methods:**

A cross-sectional diagnostic imaging study was performed. Twenty out of 50 patients were randomly selected from our index prospective cohort (IPC): a prior study evaluating dorsal tangential views (DTVs) in reducing dorsal screw penetration in internal fixation of intra-articular distal radius fractures using post-operative CT scans to quantify screw protrusion. Pre-operative CTs from this cohort were now used for 3DVP by three experienced orthopaedic trauma surgeons (supplementary video). 3DVP was compared with the corresponding post-operative CT for assessing screw lengths and incidence of dorsal penetration. The Wilcoxon Signed Ranks test was used to compare screw lengths and the Fishers’ exact for incidence of penetration.

**Results:**

Three surgeons performed 3DVP for 20 distal radius fractures and virtually applied 60 volar plates and 273 screws. Median screw length was shorter in the 3DVP when compared to IPC: 18 mm (range, 12–22) versus 20 mm (range, 14–26) (*p* < 0.001). The number of penetrating screws was 5% (13/273 screws) in the 3DVP group compared to 11% (10/91 screws) in the IPC (*p* = 0.047). Corresponding to a reduction in incidence of at least one dorsally penetrating screw in 40% of patients in the IPC group, to 18% in the 3DVP group (*p* = 0.069).

**Conclusion:**

Three-Dimensional Virtual Pre-Operative Planning (3DVP) may reduce the incidence of dorsally penetrating screws in patients treated with volar plating for intra-articular distal radius fractures.

**Level of evidence:**

II, diagnostic imaging study.

**Supplementary Information:**

The online version contains supplementary material available at 10.1007/s00068-021-01800-2.

## Introduction

Tenosynovitis and rupture of extensor tendons due to iatrogenic dorsal screw penetration have been reported as complications after open reduction and internal fixation of distal radius fractures with use of a volar plate [2–8]. Strategies to reduce the incidence of dorsal screw penetration involve the use of Dorsal Tangential Views (DTV) and 3D Fluoroscopy [3, 9–11]. These techniques have been reported to decrease dorsal penetration from at least one dorsally penetrating screw in 40% of patients with standard 2D fluoroscopic imaging to 32% with use of DTV intra-operatively, and a further reduction to 25% has been observed using—resource intensive—3D Fluoroscopy intra-operatively [1].

In recent years, three-dimensional virtual pre-operative planning (3DVP) has become increasingly available to orthopaedic trauma surgeons [12–16]. However, due to its novelty, studies on preoperative implant choice and postoperative outcomes after 3DVP augmented fracture surgery remain scarce. Two studies [17, 18] indicate that the use of 3DVP results in good reliability between observers for screw length and may result in a lower number of screws with an inappropriate length, compared to conventional pre-operative planning with plain radiographs and CT. In line with these preliminary results, pre-operative 3DVP—which only takes 30 min—may also contribute to an additional reduction in dorsal screw penetration as compared to intra-operative DTV and 3D Fluoroscopy.

We therefore conducted a cross-sectional diagnostic imaging study on 3D virtual pre-operative planning for intra-articular distal radius fractures to answer the main study question: does pre-operative 3DVP result in an additional reduction of dorsal screw penetration? Pre-operative Computed Tomography (CT) scans from our index prospective cohort [1] (IPC) were retrospectively used for 3DVP and compared to the corresponding post-operative CTs to assess potential differences in number of dorsal screw penetrations. We aimed to (1) compare 3DVP planned screws versus screws that were placed in the actual operation of the IPC (as measured on post-operative CT) in terms of (a) screw lengths; (b) appropriate screw lengths (defined as within 75–100% of distal radius diameter); and (c) dorsal screw penetration; as well as; (2) evaluate the inter-observer reliability of the chosen 3DVP screw lengths and preferred plate size between three observers.

## Materials and methods

In accordance to the Declaration of Helsinki, we retrospectively randomly selected (computerized) 20 patients from our index prospective trial [1] (IPC) of adult patients with an intra-articular distal radius fracture that was approved by our Institutional Review Board.

### Study design

The index prospective cohort (IPC) study [1], comprises of 50 patients with intra-articular distal radius fractures. They were prospectively included to evaluate the diagnostic performance of Dorsal Tangential Views (DTV) versus Three-Dimensional Fluoroscopy (3DF) to detect dorsal screw penetration after volar plating, with post-operative CT as the reference standard. All patients were surgically treated with a variable angle locking compression plate (VA-LCP, Synthes, North Ryde, NSW, Australia) for an intra-articular distal radius fracture (AO/OTA 23 Type C1-3) between May 2017 and August 2018.

**Fig. 1:**
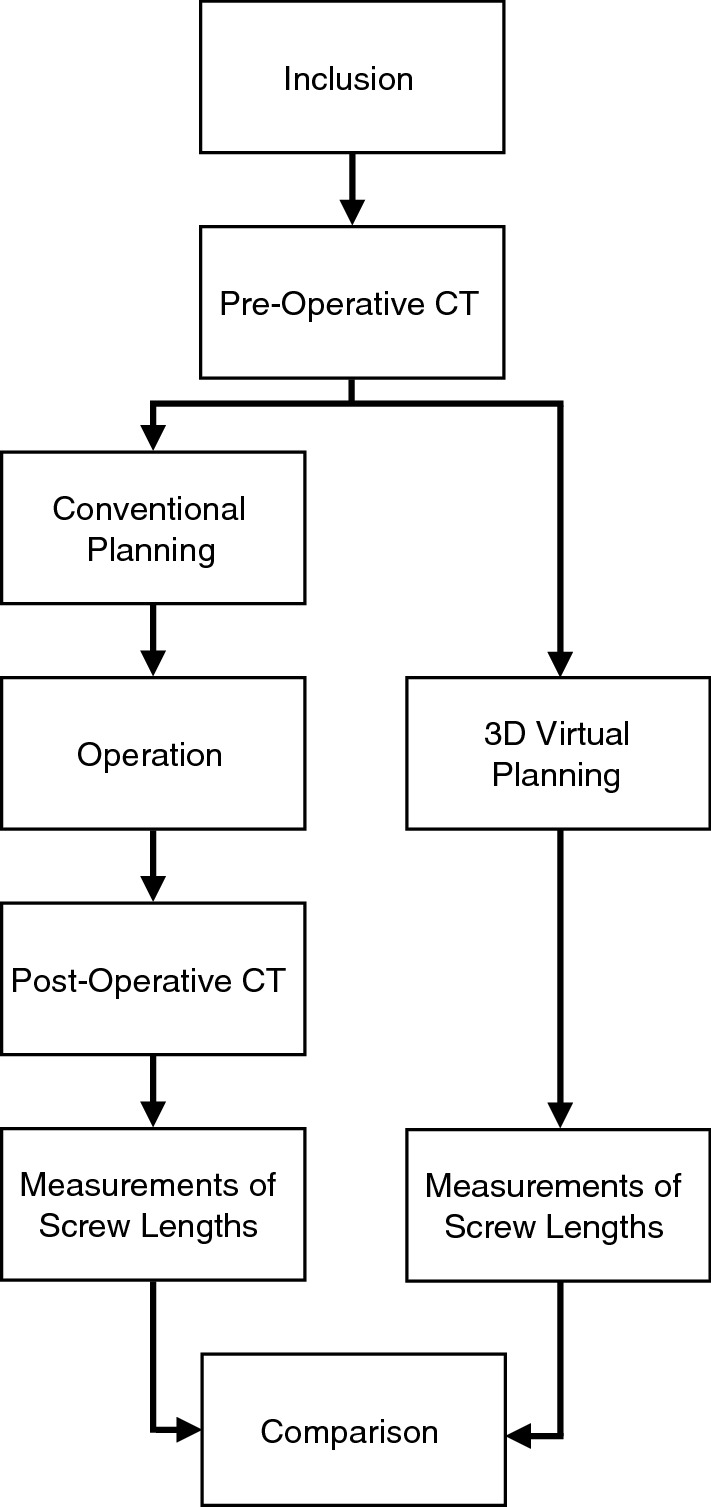
Flowchart of study design

For the purpose of our current diagnostic imaging study, 20 patients (13 females, 7 males; mean age 55.2 years (range, 29–75)) were randomly selected (computerized) from our previous study. The available pre-operative CT scan of each patient was used to perform 3DVP. Subsequently, 3DVP was compared with the corresponding post-operative CT scan of the index procedure to assess potential differences in number of dorsal screw penetrations (Fig. 1).

### CT reference standard: assessment of dorsal cortex screw penetration

**Fig. 2:**
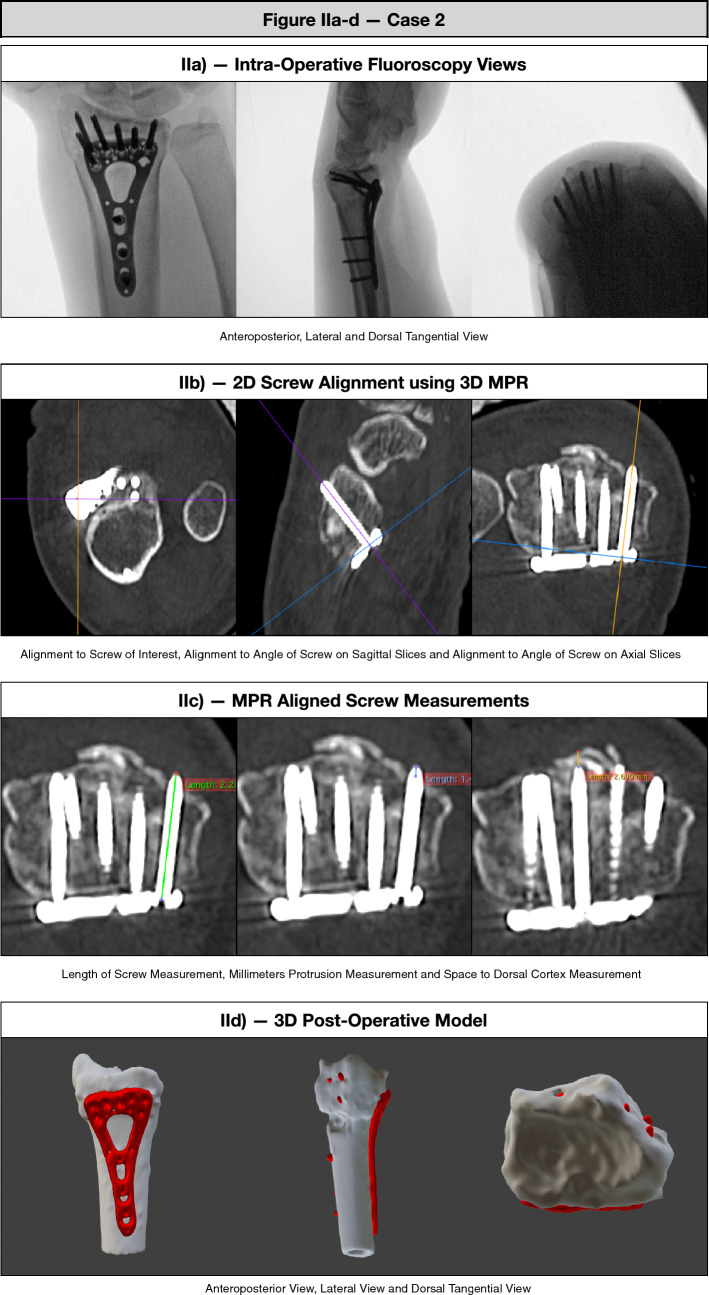
**a** Intra-operative anteroposterior, lateral and dorsal tangential fluoroscopy views; **b** alignment to screw of interest, angle of screw on sagittal slices and angle of screw on axial slices; **c** length of screw measurement, millimetres protrusion measurement and space to dorsal cortex measurement; **d** 3D model of post-operative CT scan with volar, radial and articular views

To answer the first study question, regarding the incidence of dorsal screw penetration in intra-articular distal radius fractures compared to DTV, we defined dorsal screw penetration as screws penetrating ≥ 0.5 mm, according to the definition by Sugun et al. [6]. In short: all included patients in the IPC underwent thin-slice (< 1 mm thickness) post-operative CT-scans of their wrist within 1 week (Somatom Definition AS + , Siemens, Erlangen, Germany). A multi-planar reconstruction (MPR) was created (Horos version 3.3.6). The axial view was aligned with individual screws to facilitate measurements of screw length in the correct plane and angle (Fig. 2a–d). Screw length with increments of 2 mm, was measured from insertion to tip with built-in measure tools. Dorsal screw penetration was defined as the distance in millimeters between the dorsal cortex and the protruding screw tip*.* Residual space was measured from screw tip to edge of the dorsal cortex. Appropriate screw lengths, as defined by Totoki et al. [18], are at least 75% of the diameter of the radius in the respective angle of a screw, and not longer than 100% of the diameter of the radius.

### 3D virtual pre-operative planning (3DVP)

**Fig. 3:**
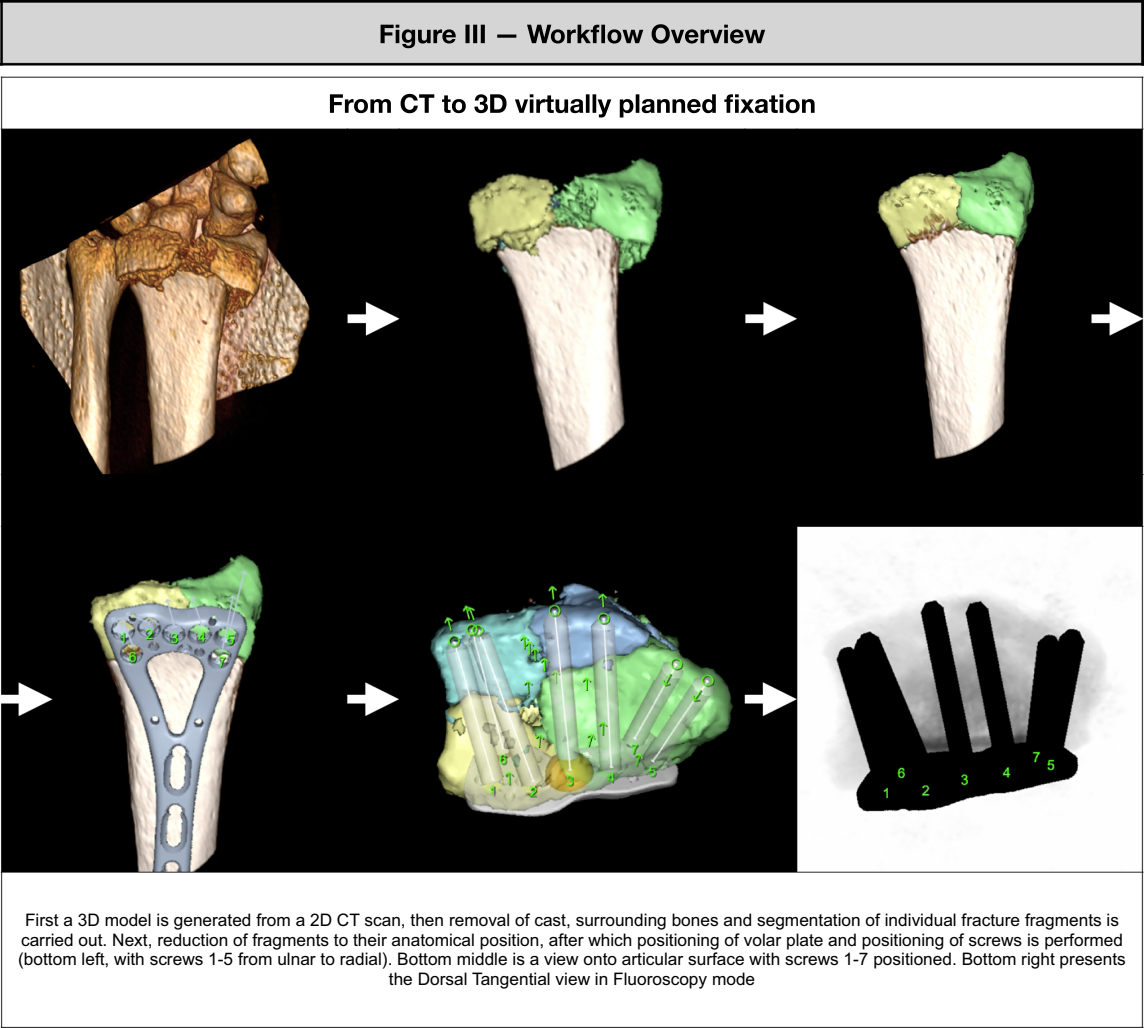
Workflow of the 3D virtual pre-operative planning, from CT to fluoroscopy representation of the 3D virtual planning

Three experienced trauma surgeons (practicing at least 5 years at consultant level) performed a series of virtual open reductions and internal fixations with volar plates on distal radius fractures from pre-operative scans of 20 patients using 3DVP software (SECTRA, Linköping, Sweden) (supplementary video). All observers in this study were blinded for screw lengths and plate size of the index procedure. 3DCT models of the distal radius were constructed from standard preoperative thin slice (< 1 mm thickness) CT scans. Subsequently, fracture fragments were aligned and reduced by virtually manipulating individual fragments in a three-dimensional space using drag-, tilt- and rotation-tools until a satisfactory anatomical reduction was obtained (Fig. 3). Surgeons then virtually applied a standard 6- or 7-head hole VA-LCP plate on the anatomically reduced distal radius. Virtual intra-operative fluoroscopy views were reconstructed to verify implant positions. Screw lengths were chosen to be their maximum length, without protruding the dorsal cortex as observed in the 3DVP model (Fig. 3). To compare screw lengths for corresponding respective screw positions, the 3DVP volar plate size (6- versus 7-head hole) was matched with the volar plate used in the actual surgery.

**Fig. 4:**
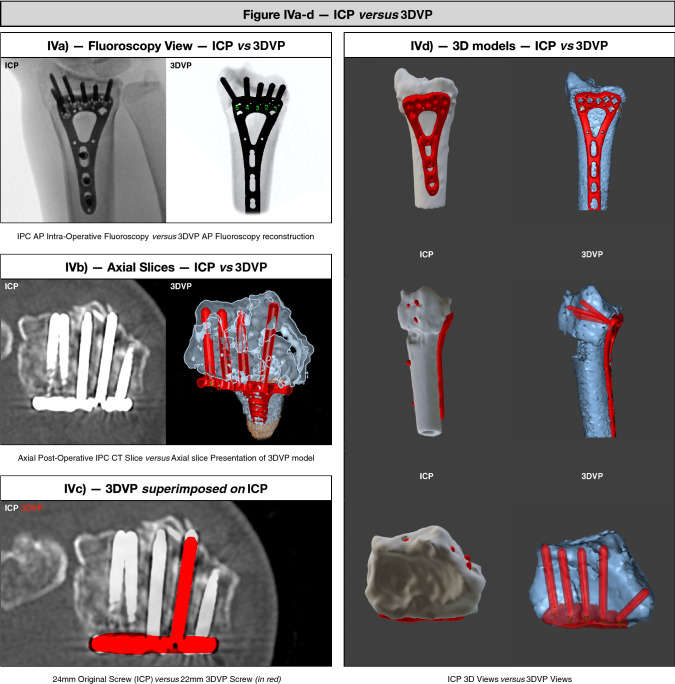
**a** AP intra-operative fluoroscopy versus AP fluoroscopy of 3DVP model; **b** axial post-operative CT slice versus axial slice presentation of the 3DVP model; **c** 24-mm original screw (ICP) versus 22 mm 3DVP Screw (in red); **d** ICP 3D views versus 3DVP views

Screw direction in degrees (within the 15-degree angle limitation of variable angle locking screws) and screw lengths (in 2-mm increments) were documented in millimeters for all screws. Screw lengths planned using 3DVP, were compared to the corresponding screws on the post-op CTs from the IPC (Fig. 4a–d). In all cases, the intra-operative reductions (e.g., gaps and step-offs < 2 mm) were nearly as good as 3DVP reductions. The five respective distal screwholes were based on anatomical landmarks corresponding to the original study [1] and defined as: (1) 4^th^ compartment on the ulnar side, (2) 3th compartment, (3) Lister’s tubercle, (4) 2^nd^ compartment on the radial side, and (5) radial styloid. 3DVP was compared with the original IPC in terms of: total screw length, residual length between tip- and dorsal cortex; *or* length of cortical breach. In addition, appropriate screw lengths (yes/no) were defined as within 75–100% of distal radius diameter [18].

### Statistical analysis

Statistical analyses were performed using the IBM SPSS software (version 27; IBM Corp., Armonk, NY, USA). The Wilcoxon Signed Ranks test was used to compare the ratio values; overall and individual screw lengths of the IPC versus 3DVP. Fishers’ exact test was used to compare percentages of dorsal screw penetration and appropriate screw lengths between 3DVP versus IPC. The Interclass Correlation Coefficient (ICC) was used to determine the inter-surgeon agreement on overall screw lengths. The ICC is scored in a 0 to 1 scale in which 0 means no correlation and 1 means perfect correlation. The Fleiss’ Kappa was used to determine the agreement between the three observers for the nominal value plate size. Pre-hoc Power Analysis (related samples binomial) revealed that 20 consecutive patients were required to show a significant decrease in incidence of dorsal screw penetration from 40 to 0% with a power of 85% and an alfa of 0.05.

## Results

### Reference standard—index prospective cohort with post-Op CT

After routine use of intra-operative DTV in our historical index prospective cohort [1], the total number of dorsally penetrating screws was 11% (10/91 screws) in the patients selected from the IPC for this study. This corresponded to 40% of patients (8/20) that had—at least—one dorsally penetrating screw on the postoperative CT scan. Two out of eight patients had two protruding screws (Table 1).

**Table 1:** Screw lengths in millimetres of the index prospective cohort with; in brackets (+) protrusion of dorsal cortex (bold), or (−) residual space till dorsal cortex

Index prospective cohort—baseline screw lengths (mm) + space to dorsal cortex (mm)											
Case	VA-LCP plate number of distal holes	Screw 1		Screw 2		Screw 3		Screw 4		Screw 5	
1	5	**24**	**(+ 0.51)**	24	(− 2.25)	24	(− 3.32)	24	(− 0.21)	20	(− 0.15)
2	5	24	(− 0.8)	24	(− 1.14)	24	(− 2.65)	24*	**(+ 1.25)**	22*	**(+ 1.45)**
3	4	18	(− 1.61)	16	(− 6.26)	16	(− 4.07)	18*	**(+ 0.75)**		
4	5	22	(− 0.23)	24	(− 3.70)	–		20	(0)	14	(− 0.58)
5	4	18	(− 3.31)	18	(− 2.05)	18	(− 2)	16	(− 0.78)		
6	4	22	(0)	22	(− 3.77)	20	(− 3.12)	18	(0)		
7	5	18	(− 3.42)	18	(− 5.17)	18	(− 5.44)	18	(− 1.36)	18	(− 1.98)
8	4	16	(− 6.18)	18	(− 6.75)	20	(− 2.39)	18	(− 2.34)		
9	5	22	(− 2.27)	22	(− 2.66)	24	(− 1.64)	24	(− 2.30)	18	(0)
10	5	22	(− 2.2)	26	(− 1.70)	24	(− 1.68)	24*	**(+ 0.72)**	18*	**(+ 0.78)**
11	5	20	(− 0.77)	20	(− 2.17)	18	(− 5.56)	14	(− 4.19)	16	(− 0.97)
12	5	20	(− 1.59)	20	(− 0.59)	20	(− 1.59)	20	(0)	18	(− 1.11)
13	4	20	(− 2.88)	18	(− 6.51)	22	(− 1.85)	18	(0)		
14	5	22	(0)	20	(− 4.35)	22	(− 4.63)	20	(− 6.29)	18*	**(+ 0.84)**
15	5	20	(− 2.74)	20	(− 6.78)	22	(− 4.13)	18	(− 1.74)	14	(− 7.13)
16	4	16	(− 2.76)	**20**	**(+ 1.62)**	18	(− 1.56)	16	(− 0.83)		
17	4	18	(− 2.09)	16	(− 2.50)	18	(− 0.83)	**14**	**(+ 1.09)**		
18	5	20	(0)	22	(0)	22	(− 1.45)	20	(− 1.64)	14	(− 1.23)
19	5	20	(0)	18	(− 3.35)	20	(− 0.80)	22	(− 1.39)	18	(0)
20	4	20	(0)	22	(− 1.05)	**22**	**(+ 0.75)**	16	(0)		

Positions of the ten dorsally penetrating screws were as follows; one screw was situated in the most ulnar position (i.e., 4th compartment), one screw in the second most ulnar screw position (i.e., 3rd compartment; EPL at risk), one screw was found to be penetrating in the central—Lister’s Tubercle—screw position (in plates with five holes), four screws in the second most radial position (i.e., 2nd compartment) and three screws were situated in the most radial position (i.e., 2nd compartment). Additionally, 1 out of 91 screws was found to be placed intra-articular in this series (distal radio-ulnar joint).

### Dorsal screw penetration: 3DVP versus IPC

Three experienced orthopaedic trauma surgeons virtually reduced 20 distal radius fractures selected from the IPC, and placed in total 60 volar plates and inserted 273 screws in the most distal row of the plate (6- or 7-head holes) in 20 unique patients in three independent rounds, using 3DVP software (SECTRA, Linköping, Sweden).

The median screw length chosen in 3DVP setting was significantly shorter compared to the actual screws used in our IPC: 18 mm (range, 12–22) 3DVP group versus 20 mm (range, 14–26) IPC (*p* < 0.001). However, the number of screws of appropriate length was similar between groups: 81% (222/273) for the 3DVP group versus 86% (78/91) for the IPC (*p* = 0.427).

The number of penetrating screws was 5% (13/273 screws) in the 3DVP group compared to 11% (10/91 screws) in the IPC (*p* = 0.047). This corresponds to a reduction in incidence of at least one dorsally penetrating screw in 40% (8/20 patients) in the IPC group, to 18% (11/60 plannings) in the 3DVP group (*p* = 0.069). A comparison of screw lengths per screw hole from ulnar to radial between IPC versus the 3DVP cohorts is presented in Table 2.

**Table 2:** Comparison of screw lengths between the IPC versus 3DVP

IPC vs 3DVP median lengths (mm)			
Screw	IPC (range)	3DVP (range)	*P* value
Ulnar			
1	20 (16–24)	18 (12–20)	0.000*
2	20 (16–26)	20 (16–22)	0.009*
3	20 (16–24)	20 (14–22)	0.030*
4	18 (14–24)	18 (12–22)	0.003*
5	18 (14–22)	14 (14–16)	0.002*
Radial			

Median lengths of screws (millimeters) with in brackets noted the range of lengths among 20 cases

*P* < 0.05 is significant

Significant *P* values are marked bold with an asterisk

**Fig. 5:**
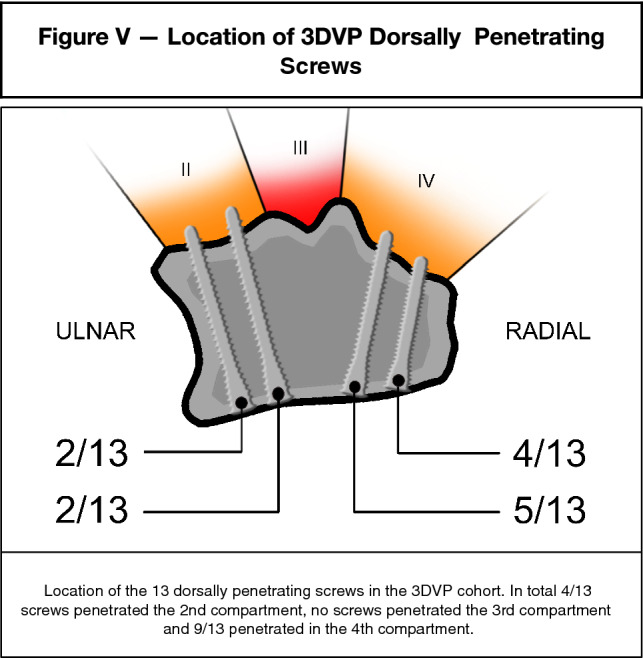
Location of the 13 dorsally penetrating screws in the 3DVP cohort. In total 4/13 screws penetrated the 2nd compartment; no screws penetrated the 3rd compartment and 9/13 penetrated in the 4th compartment

For the individual surgeons, the numbers of 3DVP dorsally penetrating screws were respectively 5% (5/91 screws), 9% (7/91) and 1% (1/91) (Table 3a–c), corresponding to the presence of at least one dorsal penetrating screw in 20% (4/20), 30% (6/20) and 5% (1/20) of patients, respectively. Further assessment of the 13 penetrating 3DVP screws, revealed that 2 (15%) of them were situated in the most ulnar position, 2 (15%) in the second most ulnar screw position, none were found in the central screw position, 5 (38%) in the second most radial position and 4 screws (31%) were situated in the most radial position (Fig. 5).

**Table 3:** a-c 3DVP Screw lengths and protrusion per individual observer

Case	VA-LCP plate number of distal holes	Screw 1	Screw 2	Screw 3	Screw 4	Screw 5
(a) 3D virtual planning—1st observer						
1	5	20	20	22	20	14
2	5	20	20	20	22	16
3	4	16	16	18	14	
4	5	20	20	–	20	**16 (+ 1.42)**
5	4	18	20	18	14	
6	4	20	20	22	16	
7	5	18	20	20	18	14
8	4	16	16	20	12	
9	5	18	22	20	22	14
10	5	20	20	20	20	14
11	5	18	18	20	18	14
12	5	20	20	20	20	14
13	4	18	18	20	14	
14	5	20	20	20	22	14
15	5	18	18	18	18	16
16	4	16	16	14	14	
17	4	16	16	18	**14 (+ 1.09)**	
18	5	18	20	20	18	14
19	5	**22 (+ 2)**	**22 (+ 0.65)**	20	20	14
20	4	**22 (+ 2)**	22	20	16	
(b) 3D virtual planning—2nd observer						
1	5	20	20	18	22	20
2	5	18	20	22	22	14
3	4	18	16	20	16	
4	5	14	22	–	20	14
5	4	12	20	**22 (+ 2)**	14	
6	4	16	20	22	16	
7	5	18	18	20	18	18
8	4	14	18	18	18	
9	5	18	22	22	22	14
10	5	18	22	22	22	12
11	5	18	20	20	**20 (+ 1.81)**	10
12	5	18	**22 (+ 1.41)**	18	20	14
13	4	12	20	20	18	
14	5	20	24	24	18	16
15	5	18	20	20	**20 (+ 0.26)**	16
16	4	12	16	18	14	
17	4	18	18	**20 (+ 1.17)**	16 (+ 3.09)	
18	5	14	22	22	20	12
19	5	10	20	20	22	16
20	4	16	22	**22 (+ 0.75)**	16	
(c) 3D virtual planning—3rd observer						
1	5	16	22	20	22	16
2	5	12	16	20	20	10
3	4	14	16	16	14	
4	5	12	18	–	18	12
5	4	12	18	20	16	
6	4	12	20	22	16	
7	5	16	16	18	16	12
8	4	16	16	18	10	
9	5	12	14	20	20	14
10	5	14	18	18	20	14
11	5	16	18	18	18	14
12	5	18	18	18	20	15
13	4	18	18	18	12	
14	5	16	18	18	18	12
15	5	18	18	20	18	16
16	4	12	16	14	12	
17	4	12	16	18	12	
18	5	16	18	20	18	**16 (+ 0.77)**
19	5	20	20	20	18	10
20	4	18	18	20	14	

Virtually planned screw lengths exceeding the length as used in our cohort are highlighted in bold

Millimeter difference between cohorts are noted in brackets next to screw length

### 3D virtual planning (3DVP): inter-observer reliability of plate size and screw lengths

In all cases, a variable angle locking plate (Synthes, Variable Angle LCP Two-Column Volar Distal Radius Plate 2.4) was used. Inter-surgeon reliability for 3DVP plate size was fair [19] (Fleiss’ Kappa of 0.221) (6- versus 7-head hole) and excellent [20] (ICC of 0.770) for screw length. Surgeons would have chosen a different size plate from what was used in the IPC, in 50% (10/20), 50% (10/20) and 30% (6/20) of cases for each respective surgeon. Seventeen out of 36 (48%) cases where a 7-head hole plate was used, surgeons preferred the smaller 6-head hole based on the 3DVP.

## Discussion

The aim of this study was to evaluate the effect of three-dimensional virtual pre-operative planning (3DVP) on the incidence of dorsal screw penetration after volar plating of distal radius fractures. This study demonstrated that 3DVP planned screw lengths were significantly shorter, as compared to the corresponding screws that were placed in clinical practice in our index prospective cohort [1] (IPC). Slightly shorter screws, however, did not result in screws of inappropriate (i.e., too short) lengths. Three-Dimensional Virtual Pre-Operative Planning (3DVP) potentially reduces the incidence of dorsally penetrating screws in patients treated with volar plating for intra-articular distal radius fractures.

This study should be interpreted in the light of strengths and weaknesses: major strength includes evaluation of 3D virtual pre-operative planning software as applied to a factual clinical scenario by experienced orthopaedic trauma surgeons (i.e., clinicians). In contrast, in studies to date in orthopaedic trauma the segmentation and planning was mostly executed on locally developed software limiting the external validation of these applications. Limitations of this study include its retrospective design with limited number of patients, need of a CT scan which may not be appropriate in more simple distal radius fractures, selection bias due to inclusion of complex intra-articular fractures only and the fact that at this stage in development 3DVP is 30 min after the initial learning curve.

The incidence of dorsal screw penetration was not reduced to zero. This may result from slight differences between the virtual reductions versus the actual post-operative reduction as observed on CT. Although, in our series all postoperative CTs from the IPC were reassessed and the intra-operative reductions were nearly as good as 3DVP reductions. Moreover, (a) the variable angle chosen (within limit of 15 degrees) in the 3DVP may deviate from the angle chosen in clinical practice in the original IPC, which could not be anticipated for due to the retrospective study design; and (b) actual plate position in the IPC may have differed from volar plate position when virtually planned. The relative importance of these factors need to be studied prospectively to further discover pearls and pitfalls of 3DVP in planning of complex trauma cases.

3DVP has the potential to further optimize preoperative preparations in orthopaedic trauma care. Inter-observer agreement for screw lengths (ICC of 0.770) in this study, is comparable to the ICC (0.860) reported by Yoshii et al. [17]. Although Totoki et al. [18], reported a reduced amount of inappropriate screw lengths—defined as a ratio between screw length and radius diameter, smaller than 0.75 or larger than 1.00—in the 3DVP group, exact numbers of dorsal screw penetration are lacking. This study has some important additions to the potential of 3DVP as mentioned in the two prior studies [17, 18]. For example, this study specifically evaluated 3DVP in the context of a relevant clinical problem—dorsal screw penetration. Furthermore, the software in this study has the ability to segment individual fragments on thin-slice CTs, compared to slicing fragments with a rough cut tool [17, 18] that does not follow pathoanatomy in the latter studies. Our contemporary 3DVP software results in improved anatomical reduction and reliable configuration of fracture characteristics to prepare for surgery. Finally, the application of 3DVP in this study is executed by surgeons, without support of an engineer, thereby strengthening external validation of results when used by other surgeons without such support.

From an educational and resident training perspective, 3DVP allows surgical trainees to accurately plan the surgery ahead of time, to visualize his/her operative steps and strategy, and to subsequently discuss with a senior. Ultimately performing the surgery according to the discussed plan, increasing the efficacy of in-theatre hands-on learning in an era where operative time for residents is increasingly scarce [21–23]. Recently it was reported that Virtual Reality benefits surgeon trainees in performing a Total Hip Arthroplasty [24]. Therefore, we feel that in addition to the potential of improving patient care, 3DVP could be an important tool to optimise surgical training. However, it should also be noted that 3DVP itself has a learning curve, and it takes time to become familiar with the software. In this study, a trained observer assisted new users in planning their first cases outside the scope of this study. Ten cases were sufficient for each observer to get familiar with the software. Planning of one case may take up to 30 min, of which a significant portion is spent on reducing fragments to their anatomic position. Artificial intelligent (AI) driven automated reduction will significantly improve the application of 3DVP software in clinical practice, as one may rightfully argue that 3DVP took as much time as the actual surgery.

A critical question however is how to convert a perfect 3DVP into a successful operation. Although this is topic of further research, we recommend showing the 3DVP planning on a screen in the operating room, with clear indication of the position and length of each individual screw. Adding a 3D print of the construct created in 3DVP could further facilitate the translation to clinical practice.

3DVP may further decrease the incidence of iatrogenic dorsal screw penetration after volar plating of distal radius fractures. Prospective studies are warranted to evaluate the accuracy of translating the preoperative 3DVP to the operative procedure itself, preferably in a randomized control trial.

## Supplementary Information

Below is the link to the electronic supplementary material.

Supplementary file1 (MP4 15378 KB)
